# Antitumor effects of high-temperature hyperthermia on a glioma rat model

**DOI:** 10.3892/ol.2014.1852

**Published:** 2014-02-04

**Authors:** HIDEFUMI TAKAGI, KAZUO AZUMA, TAKESHI TSUKA, TOMOHIRO IMAGAWA, TOMOHIRO OSAKI, YOSHIHARU OKAMOTO

**Affiliations:** 1 The United Graduate School of Veterinary Science, Yamaguchi University, Yamaguchi 753-8515, Japan; 2 Takagi Animal Clinic, Saijo, Ehime 793-0035, Japan; 3 Faculty of Agriculture, Tottori University, Tottori 680-8533, Japan

**Keywords:** high-temperature hyperthermia, glioma, apoptosis, Ki-67

## Abstract

High-temperature hyperthermia (HTH) is an established treatment option for cancer. The aim of the present study was to reveal the exact correlation between HTH at temperatures of 50–70°C and the resulting antitumor effects, using a glioma rat model. In the 60°C (T-60) and 70°C (T-70) HTH groups, tumor growth rates were significantly suppressed compared with those in the nontreatment (NT) group. In the 50°C (T-50) HTH group, tumor growth rates were not suppressed compared with those in the NT group. The numbers of terminal dUTP nick-end labeling-positive cells in tumor tissue were significantly higher in the T-50, T-60 and T-70 groups than those in the NT group. The Ki-67-positive areas were significantly decreased in the T-70 group compared with those in the NT and T-60 groups. Our data indicate that HTH at 60 and 70°C suppresses tumor growth in a glioma rat model. In particular, cell proliferation was significantly suppressed by HTH at 70°C. However, differences in the mechanism of action of HTH at 60 and 70°C were observed.

## Introduction

Hyperthermia has long been established as a treatment option for cancer, particularly for superficial types of cancer (1). Hyperthermia is used alone or as an adjunct to radiotherapy or chemotherapy (2–5). Traditional hyperthermia (41–43°C, or even lower) can synergistically enhance the therapeutic effects of radiotherapy by inducing apoptosis. In hyperthermia with thermal ablation (>60°C), direct killing of tumor cells occurs by necrosis (6,7).

High temperatures (>46°C) can directly induce cell damage, including severe protein denaturation and DNA damage (8,9). These changes are irreversible and ultimately result in cell death. Tumor cells express specific tumor-associated antigens. In high-temperature (e.g. >46°C) conditions, tumor cells swell and break into pieces, allowing antigen release. This creates a large antigen load for the generation of antitumor immunity. Although high temperatures cause severe protein denaturation, this is likely to destroy the immunogenicity of tumor cells (10–14). When thermal ablation temperatures (>60°C) are achieved, there is a high risk of shock syndrome induced by the sudden and large production of necrotic tumor material. However, a relatively low temperature range (46–55°C) can increase the proportion of apoptotic cells among the dead cells, which is likely to reduce the risk of shock syndrome (15). To the best of our knowledge, there are no previous studies which aimed to determine the correlation between temperatures of 50–70°C and antitumor effects.

Glioma is the most common brain tumor. Surgery, chemotherapy and radiotherapy form the basis of glioma treatment. However, the prognosis of patients with glioma is poor (16). The development of alternative therapies for patients with glioma is essential to improve their prognosis. Previously, it was indicated that hyperthermia can prolong the survival time and rate of patients with glioma (17). Specific reports have revealed the mechanisms of action of hyperthermia on glioma cell lines (18,19). However, mild-temperature hyperthermia was utilized in these studies.

The aim of the present study was to clarify the exact correlation between high-temperature hyperthermia (HTH) at 50–70°C and the resulting antitumor effects, using a glioma rat model. The effects of HTH were evaluated in a glioma rat model by histopathological examination.

## Materials and methods

### Treatment device

In this study, a tissue ablation device for veterinary medicine (AMTC 200; Alexon Inc., Ehime, Japan) was used. This device can regulate temperature between 50 and 70°C.

### Preparation of the glioma-bearing mouse model

F344 rats (female; 31–37 weeks old) were purchased from CLEA Japan (Osaka, Japan). The animals were maintained under conventional conditions. The use of these animals and the procedures they underwent were approved by the Animal Research Committee of Tottori University (Torrati, Japan).

9L cells were maintained in E-MEM (Wako, Inc., Osaka, Japan) containing 10% fetal bovine serum, 0.1 mg/ml neomycin, 0.05 mg/ml streptomycin and 0.05 mg/ml penicillin at 5% CO_2_ and 37°C under a humidified atmosphere in an incubator. The rats were anesthetized via inhalation of 3–5% isoflurane (Intervet, Inc., Tokyo, Japan). In total, 2.5×10^7^ cells (0.2 ml) were injected subcutaneously into the dorsal regions of each rat. Rats with tumor diameters exceeding 10 mm were used for the experiment.

### Study design

Rats (n=14) were randomized into four groups: Nontreatment (NT; n=3), 70°C (T-70) HTH (n=3), 60°C (T-60) HTH (n=4) and 50°C (T-50) HTH (n=4) groups. The tumor growth rates were calculated according to the tumor volumes (mm^3^/day). On day 0, HTH was performed for 10 min at 70, 60 or 50°C. On days 0, 3, 6, 9 and 12, the volumes of the tumor tissues were calculated by measuring the mediastinum, transverse length and depth of each tumor. The tumor tissues were removed on day 12 and fixed in 10% buffered formalin.

### Histological examination

Fixed samples were embedded in paraffin and sectioned in a routine manner. The sections were subjected to hematoxylin-eosin (HE), Ki-67 and terminal dUTP nick-end labeling (TUNEL) staining.

For Ki-67 staining, tissue sections (3 μm) on glass slides were deparaffinized, washed with ethanol and water, and soaked in phosphate-buffered saline (PBS). The sections were autoclaved with 0.01 M citrate buffer (pH 6.0) for 15 min (121°C). The sections were then washed with PBS and incubated with rabbit polyclonal anti-Ki-67 antibody (1:50; E0468, Dako, Glostrup, Denmark) for 30 min at room temperature. After washing with PBS, the sections were incubated with rat anti-IgG antibody (1:100; sc-372; Vector Laboratories, Inc., Burlingame, CA, USA) for 30 min at room temperature. The slides were washed with PBS and stained using the ABC method (PK-4000; Vector Laboratories, Inc.) for 30 min.

For TUNEL staining, tissue sections (3 μm) on glass slides were deparaffinized, washed with ethanol and water, and soaked in diluted water. TUNEL staining was performed using the *In situ* Apoptosis Detection kit (Takara Bio, Inc., Shiga, Japan) according to the manufacturer’s instructions. Ten high-power fields were randomly selected and the positive cells were counted.

### Image analysis of Ki-67 staining

Quantitative digital morphometric analysis of the Ki-67-positive area was performed. Ten randomly-selected high-power fields (magnification, ×200) were photographed for each cross section using a digital camera attached to an Olympus microscope system (Olympus Corporation, Tokyo, Japan). The color wavelengths of the copied images were transformed into digital readings using Lumina Vision software (Mitani Corporation, Tokyo, Japan), allowing for quantification of the various color wavelengths with pixels as the unit of measurement. The percentage of positive areas in the tumor tissues was calculated by dividing the total pixel area of the positive areas by the total pixel area corresponding to the entire tumor tissue in the field of view. The tumor tissues of three mice were analyzed in each group. The mean scores for 30 fields were used to determine the percentage of positive areas for each group.

### Statistical analysis

Data are expressed as the mean ± standard error of the mean. Statistical analyses were performed using one-way analysis of variance followed by the Tukey-Kramer test or the Steel-Dwass test. P<0.05 was considered to indicate a statistically significant difference.

## Results

### Tumor growth rates

**Figure 1:**
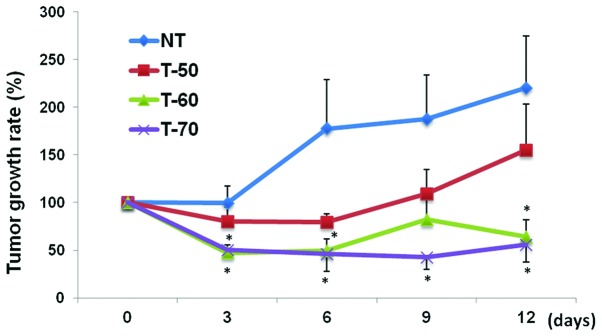
Effects of high-temperature hyperthermia on tumor growth. The tumor volume was measured on days 0, 3, 6, 9, and 12. The tumor growth rates were calculated according to the tumor volumes. The data are presented as the mean ± standard error of the mean for each group. Statistical significance was determined using the Tukey-Kramer test; ^*^P<0.05 vs. NT group.

The tumor growth rates of the various groups are shown in Fig. 1. The tumor growth rates were significantly lower in the T-70 group than those in the NT group on days 3, 6, 9 and 12 (P<0.05). The tumor growth rates were significantly lower in the T-60 group than those in the NT group on days 3, 6 and 12. The tumor growth rates were slightly, but insignificantly, lower in the T-50 group than those in the NT group. The tumor growth rates were also lower in the T-70 and T-60 groups than those in the T-50 group on days 3, 6, 9 and 12. The tumor growth rates in the T-60 and T-70 groups were similar on days 3, 6 and 12.

### Histological evaluation

**Figure 2:**
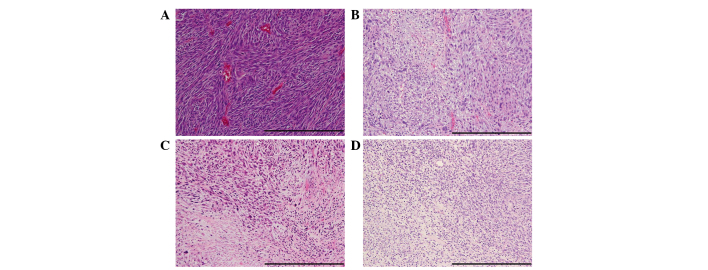
Effects of high-temperature hyperthermia on histological changes. The tumor tissue sections were stained with hematoxylin and eosin (bar, 200 μm). Data are presented for one mouse each from the (A) nontreatment, (B) 50, (C) 60 and (D) 70° high-temperature hyperthermia groups.

The results of HE staining are shown in Fig. 2. In the NT group, active cell proliferation was frequently observed. Cell proliferation was markedly suppressed in the T-70 and T-60 groups compared with that in the NT group. In addition, in the T-70 and T-60 groups, necrotic cells were widely observed. Cell proliferation was slightly suppressed in the T-50 group compared with that in the NT group, and only a few necrotic cells were observed.

### TUNEL staining

**Figure 3:**
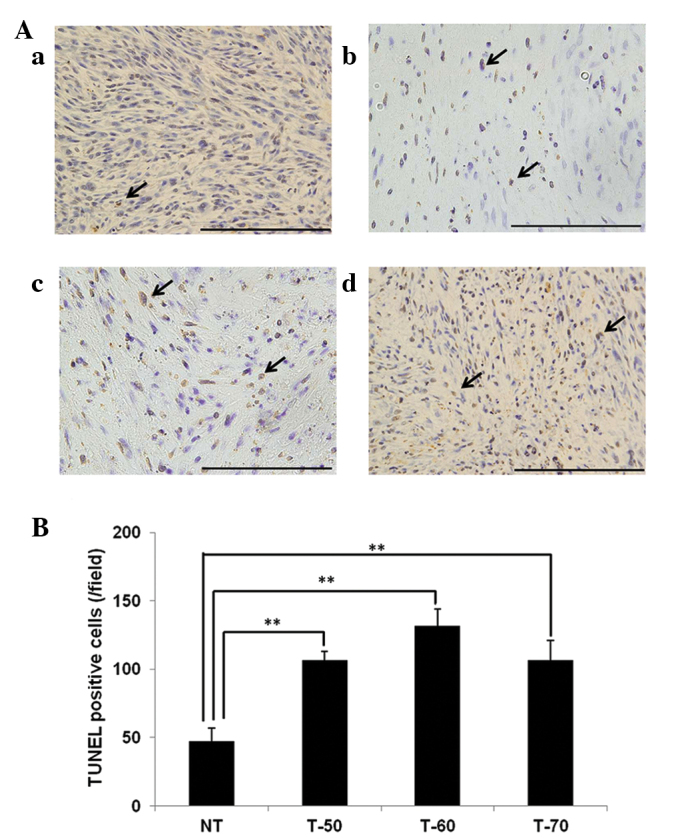
Effects of high-temperature hyperthermia on the number of TUNEL-positive cells in the tumor tissue. (A) The tumor tissue sections were stained with TUNEL (bar, 100 μm). Data are presented for one mouse each for the (a) NT, (b) T-50, (c) T-60 and (d) T-70 groups. (B) The numbers of TUNEL-positive cells were calculated. The data are presented as the mean ± standard error of the mean for each group. Statistical significance was determined according to the Steel-Dwass test; ^**^P<0.01. TUNEL, terminal dUTP nick-end labeling; NT, nontreatment; T-50, 50° high-temperature hyperthermia; T60, 60° high-temperature hyperthermia; T-70, 70° high-temperature hyperthermia.

The results of TUNEL staining are shown in Fig. 3A. The TUNEL-positive cells are denoted by arrows. The numbers of TUNEL-positive cells were significantly higher in the T-70 (106.1±14.2 cells/field), T-60 (131.4±12.4 cells/field) and T-50 groups (106.7±6.7 cells/field) than those in the NT group (47.1±9.5 cells/field) (P<0.01) (Fig. 3B).

### Ki-67 staining

**Figure 4:**
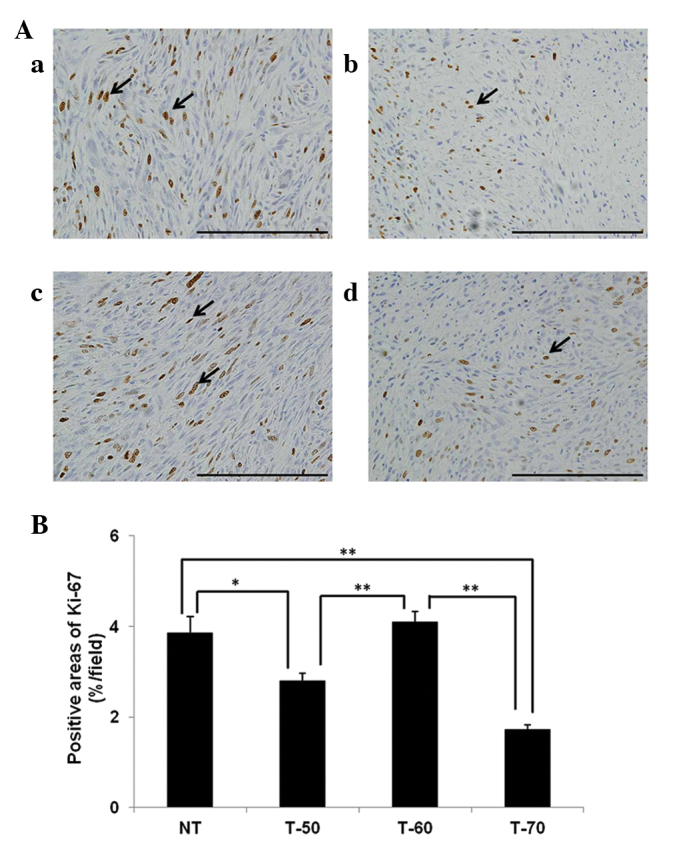
Effects of high-temperature hyperthermia on the size of the Ki-67-positive areas in the tumor tissue (bar, 100 μm). (A) Tumor tissue sections were immunohistochemically stained with Ki-67. Data are presented for one mouse each for the (a) NT, (b) T-50, (c) T-60 and (d) T-70 groups. (B) Proportions of Ki-67-positive areas were calculated. The data are presented as the mean ± standard error of the mean for each group. Statistical significance was determined according to the Steel-Dwass test; ^*^P<0.05; ^**^P<0.01. NT, nontreatment; T-50, 50° high-temperature hyperthermia; T60, 60° high-temperature hyperthermia; T-70, 70° high-temperature hyperthermia.

The results of Ki-67 stains are shown in Fig. 4A. Ki-67-positive areas are denoted by arrows. The Ki-67-positive area was significantly smaller in the T-70 group (1.7±0.1%/field) than that in the NT (3.9±0.4%/field) and T-60 groups (4.1±0.2%/field) (P<0.01). The Ki-67-positive area was significantly smaller in the T-50 group (2.8±0.2 %/field) than in the NT and T-60 groups (P<0.05, vs. NT; P<0.01 vs. T-60) (Fig. 4B).

## Discussion

In the present study, the antitumor effects of HTH were evaluated using a glioma rat model. HTH at 60 and 70°C significantly suppressed tumor growth. Previously, specific reports indicated that HTH has potency as a treatment for melanoma in experimental models (23,24). Li *et al* (24) previously described that local HTH (≥50°C) inhibited tumor growth and stimulated a favorable antitumor immune response in a malignant melanoma model. However, the authors did not investigate the correlation between higher temperatures (>60°C) and antitumor effects.

Studies of hyperthermia have focused on two commonly applied strategies, conventional hyperthermia at mild temperatures (42–45°C) (1,20,21) and ablation therapy at high temperatures (>70°C) (22). To the best of our knowledge, no study has examined the difference in antitumor effects between mild (42–45°C) and high temperatures (>70°C) under the same conditions as performed in the present study. Necrotic cells were more commonly observed in the T-60 and T-70 groups than in the NT groups. Previous reports indicated that HTH directly induced cell damage and necrosis (6,7). In the T-50, T-60 and T-70 groups in the present study, the numbers of TUNEL-positive cells in tumor tissues were significantly increased compared with those in the NT group. This finding indicates that relatively low temperatures induce apoptosis (15). Our data also indicate that HTH at 50–70°C induces necrosis and apoptosis in a glioma rat model.

Ki-67 is a cell proliferation marker that is detected during all active phases of the cell cycle, but is absent in resting cells (25). Ki-67 expression increases during S phase until mitosis, when its expression is maximal. Following cell division, cells in G1 phase exhibit decreased Ki-67 expression until they reenter the S phase when the level of Ki-67 increases again (26). Ki-67 expression is also useful for diagnosing and assessing the grade of tumors in the central nervous system (27). The Ki-67-positive areas were significantly smaller in the T-50 and T-70 groups than in the NT groups. Our data also indicate that temperatures exceeding 70°C sufficiently suppress cell proliferation. Following suppression of cell proliferation, apoptosis may be induced in circumferential areas. The Ki-67-positive area was significantly smaller in the T-70 group than that in the T-60 group, although the tumor growth rates in these groups were equally decreased. We cannot explain this difference, however, one possible explanation may be that there are differences in the percentages of apoptotic cells, as the T-60 group had significantly more TUNEL-positive cells than the other groups. HTH at 60°C may suppress tumor growth by inducing apoptosis more significantly than HTH at other temperatures. Further studies, including those of other molecules associated with apoptosis, are required to clarify this point.

In conclusion, HTH at temperatures exceeding 60°C suppressed tumor growth in a glioma-bearing rat model. In addition, HTH at 50–70°C induced necrosis and apoptosis in a glioma rat model. Further studies is required to clarify the differences in the mechanisms of action for HTH at 60 and 70°C.
